# M6A regulator methylation patterns and characteristics of immunity in acute ST-segment elevation myocardial infarction

**DOI:** 10.1038/s41598-023-42959-5

**Published:** 2023-09-21

**Authors:** Jingqi Yang, Qing Shangguan, Guobo Xie, Ming Yang, Guotai Sheng

**Affiliations:** Department of Cardiovascular Medicine, Jiangxi Provincial People’s Hospital, The First Affiliated Hospital of Nanchang Medical College, 152 Aiguo Road, Nanchang, China

**Keywords:** Computational platforms and environments, Gene regulatory networks

## Abstract

M6A methylation is the most prevalent and abundant RNA modification in mammals. Although there are many studies on the regulatory role of m6A methylation in the immune response, the m6A regulators in the pathogenesis of acute ST-segment elevation myocardial infarction (STEMI) remain unclear. We comprehensively analysed the role of m6A regulators in STEMI and built a predictive model, revealing the relationship between m6A methylations and the immune microenvironment. Differential analysis revealed that 18 of 24 m6A regulators were significantly differentially expressed, and there were substantial interactions between the m6A regulator. Then, we established a classifier and nomogram model based on 6 m6A regulators, which can easily distinguish the STEMI and control samples. Finally, two distinct m6A subtypes were obtained and significantly differentially expressed in terms of infiltrating immunocyte abundance, immune reaction activity and human leukocyte antigen genes. Three hub m6A phenotype related genes (RAC2, RELA, and WAS) in the midnightblue module were identified by weighted gene coexpression network analysis, and were associated with immunity. These findings suggest that m6A modification and the immune microenvironment play a key role in the pathogenesis of STEMI.

## Introduction

Acute ST-segment elevation myocardial infarction (STEMI) is a common disease caused by coronary artery plaque rupture, thrombus blocking blood vessels, and finally causing acute myocardial ischaemic injury, which has become one of the main threats to human health^1^. With the continuous advancement of medicine, after effective interventional surgery and drug treatment, the early mortality rate of STEMI has been significantly reduced, but the overall mortality rate is still high, and adverse cardiovascular events after acute myocardial infarction are one of the main factors causing death of patients. The possible reason is that after myocardial infarction, necrotic cardiomyocytes release danger signals, activating innate immune pathways and triggering an intense inflammatory response^2,3^, and activation of innate immune pathways leads to a stronger inflammatory response and recruitment of leukocytes, thereby aggravating myocardial injury^4,5^. Therefore, accurate identification of the immune microenvironment characteristics of each acute myocardial infarction patient may reveal its key pathological mechanism, and the use of new immune-related therapy for patients with different immune characteristics, can effectively reduce mortality and the occurrence of adverse cardiovascular events..

Currently, over 170 different types of RNA modifications have been reported, including m6A methylation, 5-methylcytosine methylation, and N1-methyladenine methylation. Among them, m6A methylation is the most prevalent and abundant in mammals^6,7^. The m6A methylation refers to the methylation of the 6th N position of adenine in the RNA molecule, which is jointly regulated by methyltransferases (writers), demethylases (erasers) and methylation recognition proteins (readers)^8^. The biological process of m6A methylation affects the splicing, transcription, translation and degradation of mRNA, thereby regulating cell differentiation, immunity and metabolism^9,10^. Recent studies have shown that m6A methylation participates in the pathogenesis, progression and treatment of multiple tumours by modulating immune responses^11^. For example, across cancers, m6A regulators may be important factors in the phenotypic modification of immune-related genes, thereby affecting tumour microenvironment characteristics^12^. In addition, the expression of m6A methylation regulatory factors was positively correlated with the degree of tumour stem cells and played an important role in anticancer drug sensitivity^13^. Although there have been many studies on the regulatory role of m6A methylation in the immune response, the m6A regulators in the pathogenesis of STEMI remain unclear.

In our research, we systematically assessed the patterns of m6A methylation regulators in STEMI from the GEO database and built a predictive model, revealing the relationship between m6A methylations and the immune microenvironment. In addition, the immune characteristics and biological functions of different STEMI subtypes were analysed. The results of our study can enhance insight into the pathogenesis of STEMI from a new perspective.

## Materials and methods

### Data acquisition

The datasets of GSE59867 and GSE48060 used in our study were obtained from the Gene Expression Omnibus (GEO) database (http://www.ncbi.nlm.nih.gov/geo/). GSE59867, based on the GPL6244 platform, was published by Maciejak et al.^14^ and includes 111 peripheral blood samples from patients with STEMI at admission (STEMI group) and 46 patients with stable coronary artery disease (control group). The baseline clinical characteristics of the STEMI and control groups are summarized in Supplementary 1. Compared with the control group, the proportion of hypercholesterolemia was lower (P < 0.001), and the left ventricular ejection fraction measured on admission was significantly higher (P < 0.001) in the STEMI group.

GSE48060, based on GPL570, was used as the independent external validation set and included blood samples from 21 control and 31 AMI patients. The baseline clinical characteristics are also shown in Supplementary 1. There were significant differences in known clinical risk factors between the control group and AMI patients, including BMI, history of hypertension and the history of smoking (P < 0.05). However, therewere no differences in other risk factors between the two groups, such as total cholesterol, high -density lipoprotein, low density lipoprotein, history of diabetes, CAD family history and drug history.

The normalize between arrays function in the limma package^15^ was used to normalize the gene expression profiles. Probes corresponding to multiple genes were removed. If multiple probes were used to detect a gene, the expression level of the gene was calculated by the average expression of all probes. Then, the original expression matrix was obtained.

### Alteration analysis of m6A regulators between the STMI and control groups

Twenty-four m6A methylation regulators were based on published literature^16–19^. The differential expression of m6A methylation regulators between the STEMI group and the control group was analysed using the Wilcoxon rank sum test. Correlations among m6A RNA methylation regulators were determined using the “corrplot” package in R software, and a P-value < 0.05 was considered to indicate statistical significance. The STRING database (https://string-db.org/) was used to construct the protein–protein interaction (PPI) network of 24 m6A regulators. The "RCircos" package was used to display the distribution of 24 m6A methylation regulators on chromosomes.

### Development of a m6A gene signature for STEMI diagnosis

Least absolute shrinkage and selection operator (LASSO) regression was used to select the best predictive features among 24 m6A methylation regulators. LASSO was performed by the ‘glmnet’ package (https://CRAN.R-project.org/package=glmnet), and after 10 trials of fivefold cross-validation, the optimal model parameter λ was calculated^20^. The m6A methylation regulators with nonzero LASSO regression coefficients were included in multivariate logistical regression. Forest plots for multivariate logistical analysis were performed using the R package "ggplot2", with statistical significance at p < 0.05. The final significant variables were the key m6A methylation regulators.

The random was set seed to 123, and the GSE59857 dataset was divided into a training set and a validation set at a ratio of 7:3. Receiver operating characteristic (ROC) curve analysis was utilized to determine the diagnostic effectiveness of combining the key m6A methylation regulators in discriminating STEMI from control patients in the training set and validation set. This classifier was also validated on an independent external dataset (GSE48060) and real-time quantitative polymerase chain reaction (qPCR). We then constructed a nomogram model using the 'rms' package in R to predict the risk of STEMI based on the selected key m6A methylation regulators. In addition, the accuracy of the nomogram model was evaluated by calibration curve and decision curve analysis (DCA).

Whole blood samples were collected from five STEMI patients and five normal patients for qPCR to confirm the results. The duration of chest pain in STEMI patients was less than 12 h, and emergency percutaneous coronary intervention was performed. The study was approved by the Ethics Committee of Jiangxi Provincial People’s Hospital, and all patients signed informed consent forms. All patient samples were processed to isolate peripheral blood mononuclear cells (PBMCs) immediately after collection and stored at − 80 °C before RNA extraction. After the samples were pretreated, RNA was extracted using TRIzol reagent (Invitrogen), and qPCR was performed. Total RNA was reverse transcribed into complementary DNA by a qPCR real-time kit (Invitrogen) following the manufacturer's instructions. Relative gene expression was analysed by the 2^−ΔΔCT^ method with normalization to ACTB (internal reference gene). All primers used in this study are shown in Supplementary 1.

### Correlation analysis between m6A methylation regulators and the immune microenvironment

The immune microenvironment was evaluated by immune infiltration, immunoreactive activity and HLA family gene expression. The CIBERSORT algorithm was used to calculate immune cell infiltration in the GSE59867 dataset^21^. We conducted single-sample gene-set enrichment analysis (ssGSEA) to estimate the activity of immune reactions^22^. The infiltrating immunocyte gene-sets and the immune reaction gene-sets were downloaded from the ImmPort database (http://www.immport.org)^23^. The expression of HLA family genes in different patients was analysed in the GSE59867 dataset. The correlation between m6A methylation regulators and the immune microenvironment was calculated according to Spearman correlation analysis and using the “ggplot2” package in R for visualization.

### Consensus clustering analysis of m6A methylation patterns

Consensus clustering analysis was performed to identify diverse m6A methylation subtypes utilizing the “ConsensusClusterPlus” package on the basis of the mRNA expression profiling of 24 m6A methylation regulators^24^. The consensus clustering algorithm runs the above steps 1000 times to guarantee the robustness of clustering. Principal component analysis was performed to validate the 24 m6A methylation regulator expression subtypes. The differences in the expression of m6A methylation regulators and immune characteristics between distinct modification patterns were compared by the Kruskal–Wallis test.

### Biological enrichment analysis for the two m6A patterns

The "h.all.v7.5.symbols" and "c2.cp.kegg.v7.5.symbols" gene sets were downloaded from the Molecular Signatures Database (MSigDB), and then the expression matrix of different subgroups was used to calculate the pathway activation scores by gene-set variation analysis (GSVA)^25^. The scores of pathway activation in different subtypes were also compared also using the "limma" package, with the cut-off criterion at P < 0.05. The results of enrichment analysis were visualized by GraphPad Prism software (version 9.0) and the R package 'clusterProfiler'.

### Identification of m6A modification pattern genes

The m6A regulator meditated genes with P < 0.00001 were considered differentially expressed genes by the ‘limma’ package^15^. GSEA was performed on m6A regulator- meditated genes, with a P value < 0.05 as the cut-off criterion, and visualized by the "ClusterProfiler" package.

The WGCNA package^26^ was used to construct a weighted gene coexpression network, identify the coexpressed gene modules, explore the correlation between the gene network and subtypes, and investigate hub genes in the network. The STRING database was used to construct a PPI network of key modules, and we used “Cytoscape” for visualization and analysis^27^.

### Ethics approval and consent to participate

The study was conducted in accordance with the principles of the Declaration of Helsinki, and the study protocol was approved by the ethics committee of Jiangxi Provincial People’s Hospital.

## Results

### Expression level of m6A RNA methylation regulators between STEMI and control samples

**Figure 1:**
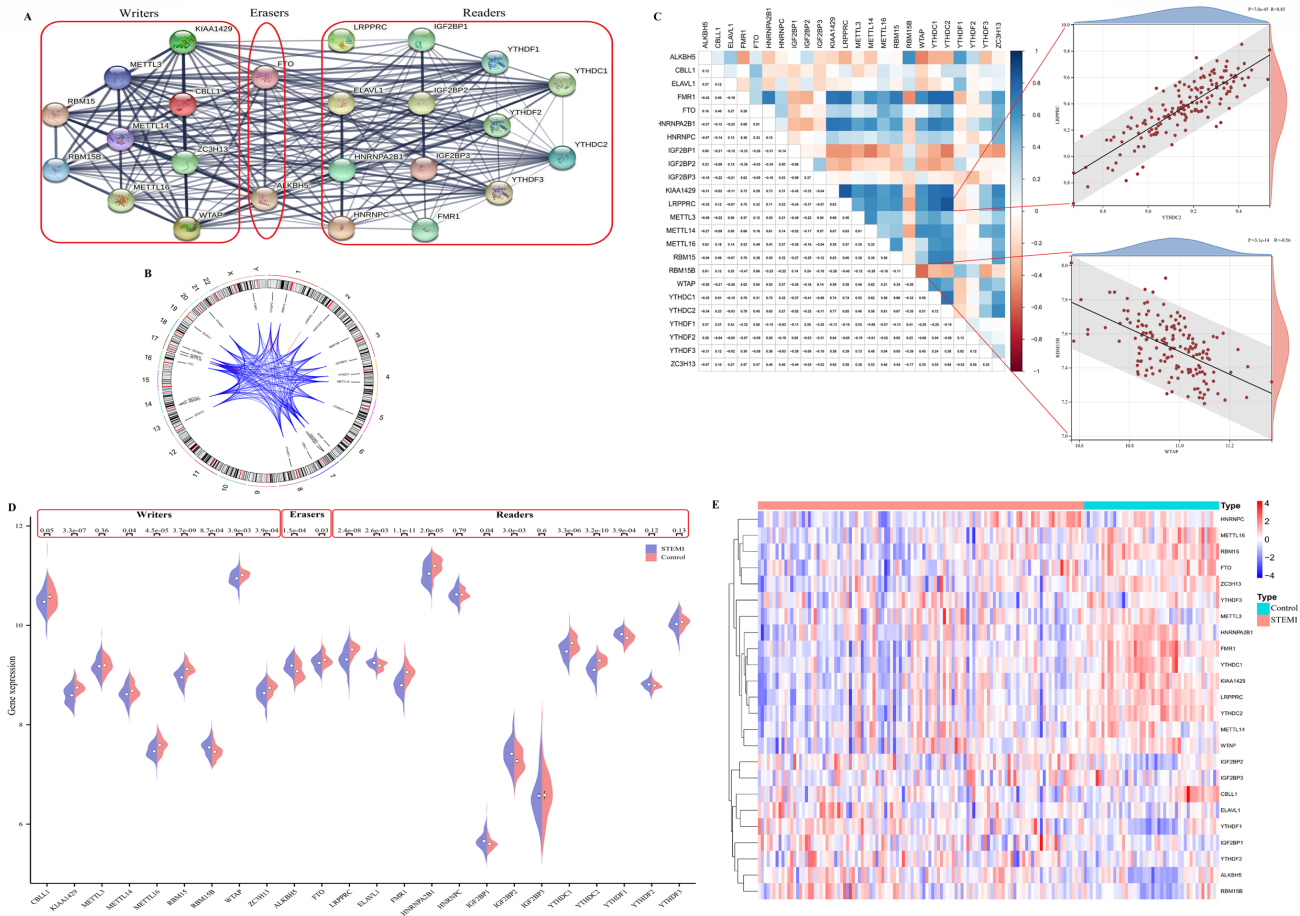
Landscape of the 24 m6A regulators in STEMI. (**A**) The composition of 24 m6A methylation regulators and protein‒protein interactions. (**B**) Chromosome separation map of 24 m6A methylation regulators. (**C**) Correlation heat-map of 24 m6A methylation regulators. The two scatter plots represent the strongest positive and negative correlations between m6A methylation regulators. (**D**) The expression of the m6A methylation regulator between the STEMI and control groups. (**E**) Heatmap of 24 m6A methylation regulators in the samples.

We downloaded the gene expression profiling data of GSE59867 from the GEO database for subsequent analysis. We analysed a set of 24 well-known m6A regulators (9 writers, 2 erasers and 13 readers) to identify different patterns of m6A methylation. To analyse the overall expression of 24 m6A regulators in STEMI, we analysed their regulatory interactions (Fig. 1A) and chromosomal localization (Fig. 1B). Surprisingly, we noticed that m6A regulators of the same type showed a strong correlation (Fig. 1C). Correlation analysis revealed that, the reader YTHDC2 exhibited the most significant positive correlation with the reader LRPPRC, whereas the writers WTAP and RBM15B were the most negatively correlated (Fig. 1C). Next, we used the Wicoxon rank sum test to compare the differences in m6A regulators in the STEMI group and the control group. The results showed that 18 of the 24 m6A regulators were statistically significant (P < 0.05), and there was no significant difference in the expression of the writers CBLL1 and METTL3 or the readers HNRNPC, IGF2BP3, YTHDF2, and YTHDF3 (P > 0.05) (Fig. 1D). The heatmap of the 24 m6A methylation genes were shown in Fig. 1E.

### Construction of the clinical prediction model based on m6A methylation regulators

**Figure 2:**
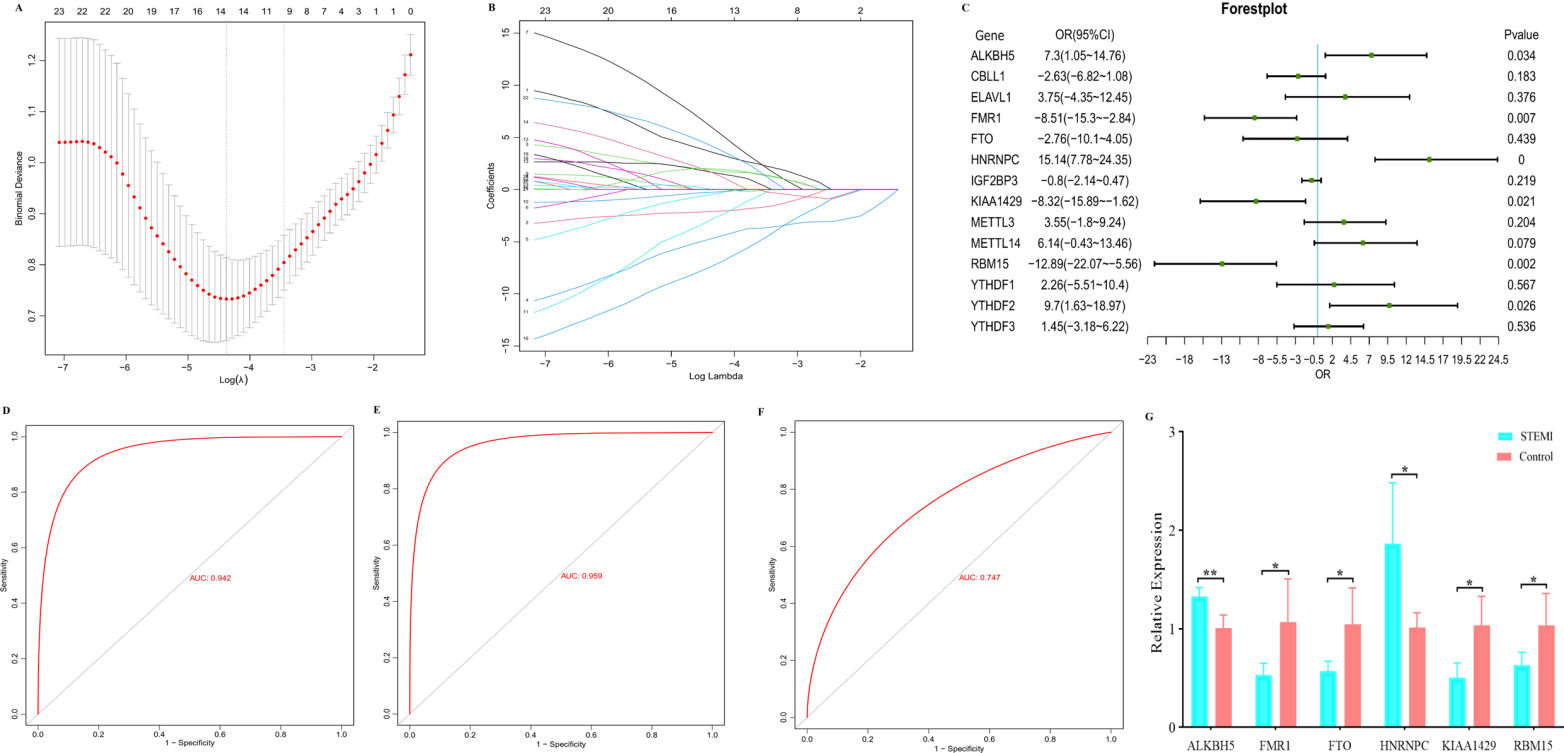
Construction of a diagnostic model to distinguish STEMI and control samples. (**A**) LASSO algorithm to screen 24 m6A methylation regulators. (**B**) Tenfold cross-validation for the coefficients of 24 m6A methylation regulators in the LASSO model. (**C**) Multivariate analysis of the nonzero coefficients in the LASSO model. (**D**) ROC curve of the training set. (**E**) ROC curve of the validation set. (**F**) ROC curve of the external validation set (GSE48060). (**G**) qPCR validation of the m6A candidate genes between STEMI and normal controls. P values were calculated using a two-sided unpaired Student’s t test.

**Figure 3:**
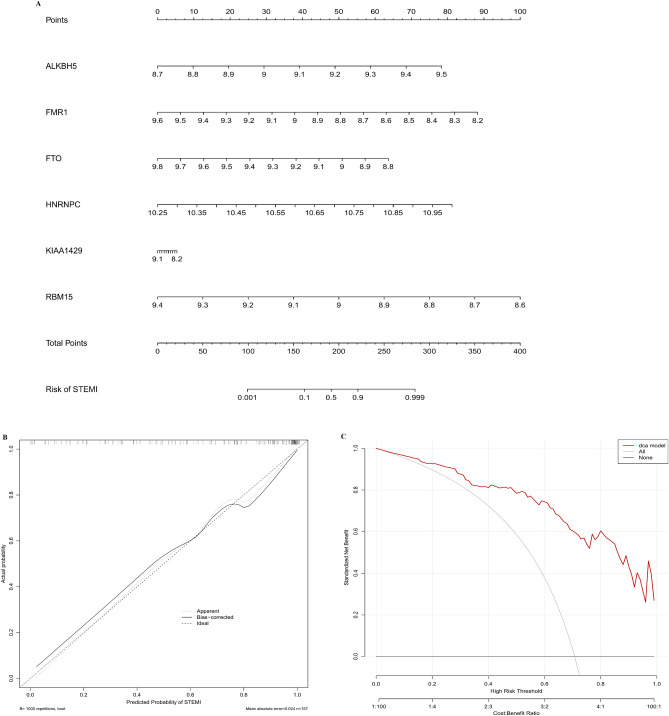
Construction of a nomogram model to predict the risk of SETMI. (**A**) The nomogram model of 6 candidate m6A methylation regulators. Total points are obtained by incorporating the corresponding points of 6 candidate m6A methylation regulators on the point scale. (**B**) The predicted probability of the nomogram model in the calibration curve. (**C**) The decision curve analysis demonstrated that the nomogram model may benefit STEMI patients. The construction and evaluation of the nomogram model were completed in the GSE93101 dataset.

To better investigate the role of m6A methylation regulators in the pathogenesis of STEMI, we established a nomogram of m6A regulators through a series of bioinformatics algorithms. First, after screening out 14 m6A methylation regulators with nonzero coefficients by LASSO regression (Fig. 2A,B), the remaining m6A methylation regulators were then included in multivariate regression analysis. Multivariate logistic regression analysis showed that ALKBH5, FMR1, HNRNPC, KIAA1429, RBM15 and YTHDF2 were independent risk factors for STEMI (Fig. 2C). Then, we performed ROC analysis based on the feature models of the 6 m6A candidate genes and found that the area under the curve (AUC) was 0.942 in the training set (Fig. 2D) and 0.959 in the validation set (Fig. 2E). We also performed validation on an additional independent external validation set (GSE48060) with an AUC of 0.747 (Fig. 2F). In addition, the qPCR results showed that the six m6A candidate genes were differentially expressed between the STEMI group and the control group (P < 0.05) (Fig. 2G). This result indicated that m6A regulators play a critical role in the occurrence and progression of STEMI, and can effectively distinguish STEMI and control samples. Finally, a nomogram model of 6 candidate m6A regulators was created according to the gene expression profiling data of GSE59867, and it was used to predict the risk of SETMI patients (Fig. 3A). In the calibration curve, the predicted probability of STEMI was consistent with the actual probability, indicating that the prediction of the nomogram model was accurate (Fig. 3B). Moreover, the DCA curve also confirmed that the nomogram model may benefit STEMI patients (Fig. 3C).

### M6A methylation regulators were associated with the immune microenvironment in STEMI

**Figure 4:**
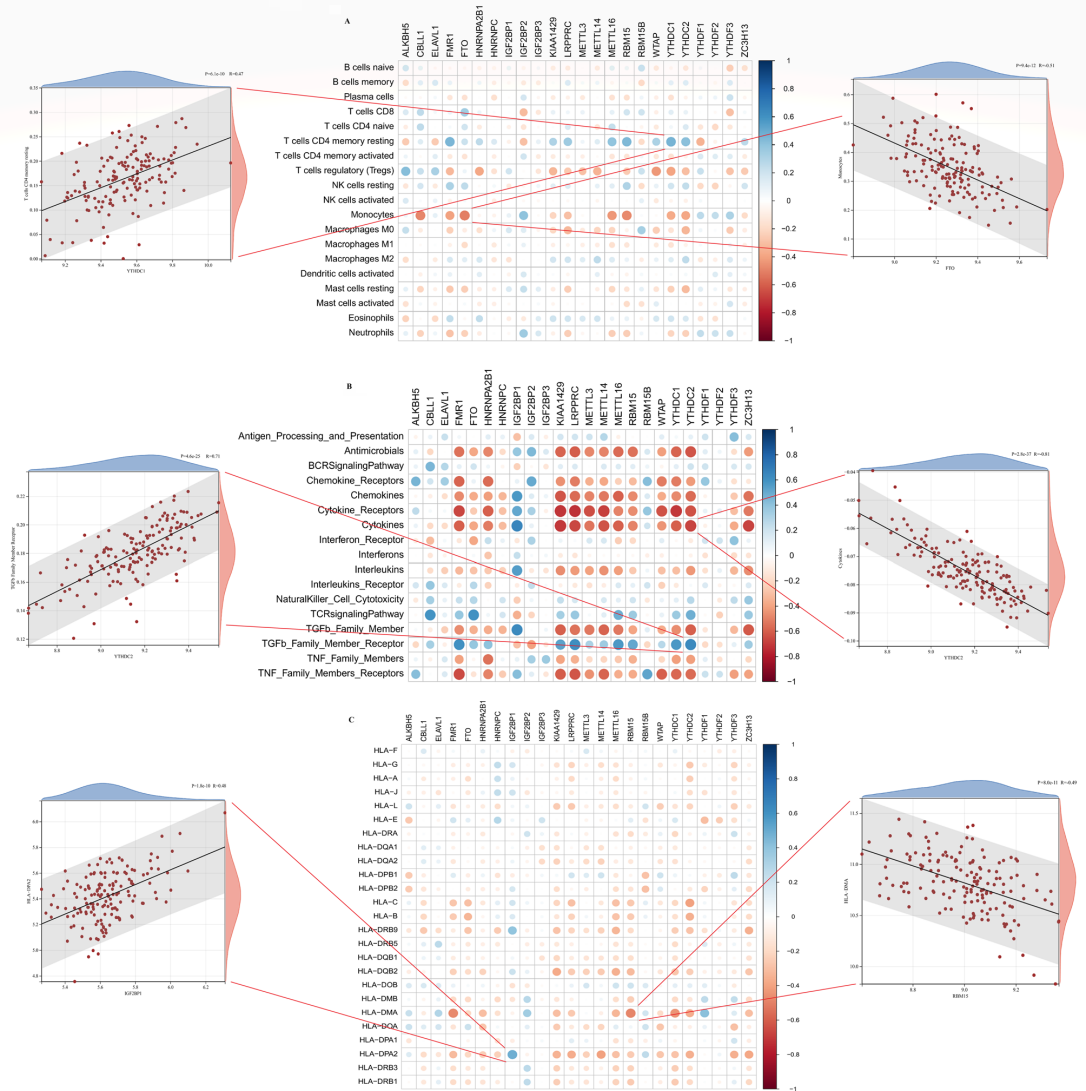
The correlation between 24 m6A methylation regulators and the immune microenvironment in STEMI. (**A**) Correlation heatmap between 24 m6A methylation regulators and immune cell infiltration. The two scatter-plots represent the strongest positive and negative correlations between m6A methylation regulators and immunocytes, respectively. (**B**) Correlation heatmap between 24 m6A methylation regulators and immune reaction activity. The two scatter plots represent the strongest positive and negative correlations between m6A methylation regulators and immune reaction activity, respectively. (**C**) Correlation heatmap between 24 m6A methylation regulators and human leukocyte antigen genes. The two scatter-plots represent the strongest positive and negative correlations between m6A methylation regulators and human leukocyte antigen genes, respectively.

To elucidate the underlying mechanism between m6A methylation regulators and immune characteristics, correlation analysis of m6A methylation regulators with immune cell infiltration, immune reaction gene-sets and human leukocyte antigen (HLA) genes was performed. The CIBERSORT algorithm was used to analyse the abundance of 22 immune cells, and found that most infiltrating immunocytes were differentially expressed between STEMI and control samples (Supplementary Fig. 1A). After excluding immune cells that were not expressed in all samples, correlation analysis revealed that infiltrating immune cells were moderately associated with m6A modulators (Fig. 4A). Among these immune cells, a positive correlation was found between YTHDC1 and resting memory CD4 T cells, while FTO was most negatively correlated with monocytes (Fig. 4A). Similarly, we analysed immune reactivity and HLA genes in STEMI and control patients. Supplementary Fig. 1B demonstrates that most of the immune response expression was significantly different between the STEMI and control groups, except for the B-cell receptor (BCR) signalling pathway. Moreover, most m6A regulators were significantly correlated with immune responses, among which YTHDC2 was positively correlated with TGFb family member receptors and negatively correlated with cytokines (Fig. 4B), suggesting that immune dysregulation in STEMI was regulated by m6A methylation. For the HLA genes, only a few were significantly changed in the STEMI group (Supplementary Fig. 1C). Correlation analysis showed that HLA weakly correlated with m6A regulators. IGF2BP1 was most positively correlated with HLA-DPA2, and RBM15 was most negatively correlated with HLA-DMA (Fig. 4C).

### Identification of m6A methylation subtypes mediated by 24 m6A methylation regulators

**Figure 5:**
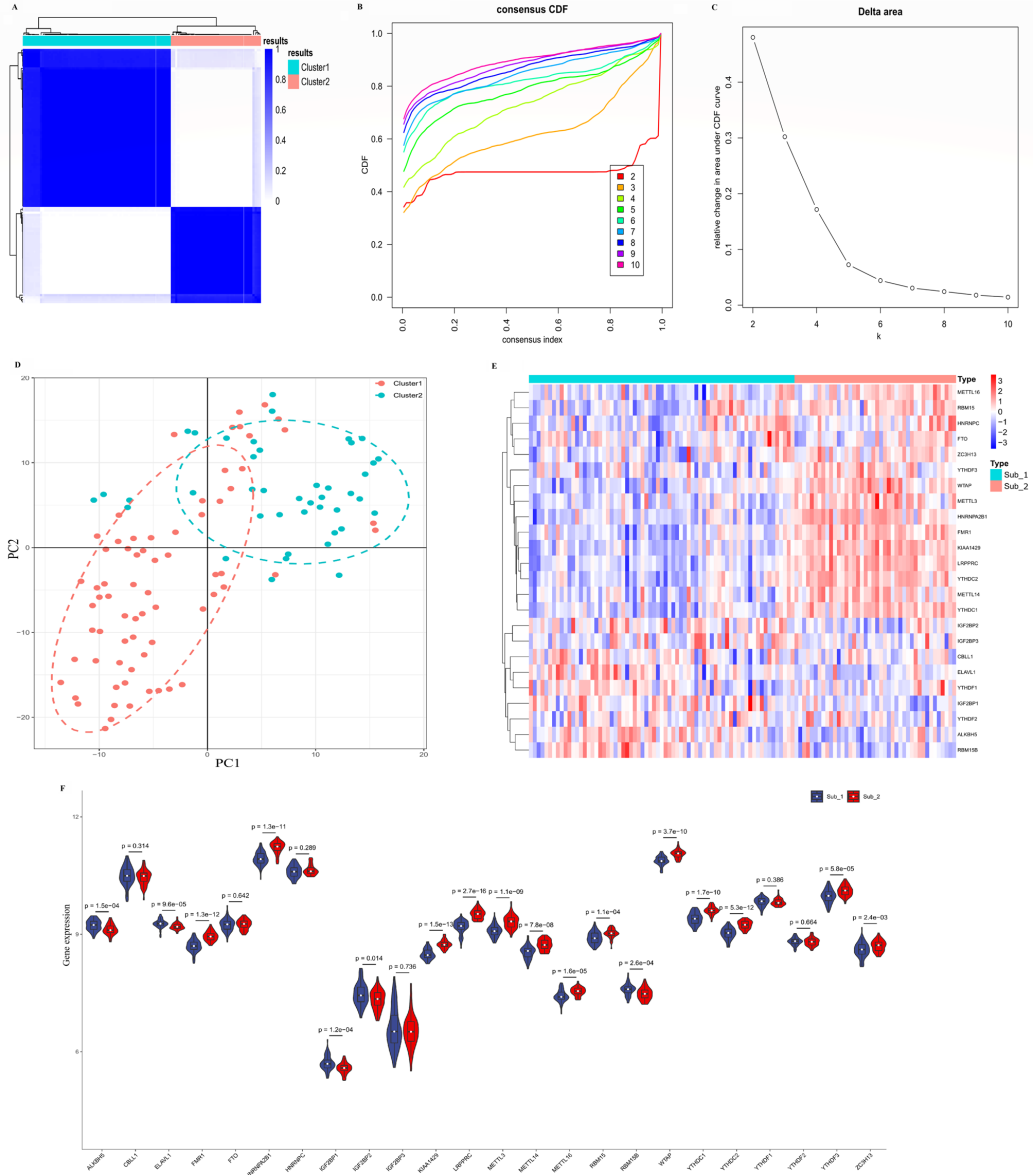
Identification of m6A methylation subtypes by unsupervised consensus clustering. (**A**) Consensus clustering matrix of the 24 m6A methylation regulators for k = 2. (**B**) Consensus clustering cumulative distribution function for k = 2–10. (**C**) Relative change in the area under the cumulative distribution function curve for k = 2–10. (**D**) Principal component analysis for the two distinct subtypes of STEMI. (**E**) Heatmap of 24 m6A methylation regulators between the two distinct subtypes in the STEMI samples. (**F**)The expression of the m6A methylation regulator in the two distinct subtypes of STEMI.

To investigate the m6A RNA methylation pattern in STEMI, we performed unsupervised consensus clustering analysis based on the expression of 24 m6A-regulated genes in STEMI samples, and two distinct subtypes of STEMI were identified according to the cumulative distribution function (Fig. 5A–C). Among them, Subtype 1 contained 69 cases, and Subtype 2 contained 42 cases. PCA showed that 24 m6A regulators could distinguish the majority of the STEMI samples in the two m6A patterns (Fig. 5D). Eighteen of the 24 m6A regulators showed marked differences between the two m6A subtypes (Fig. 5E,F), which also indicates the existence of diverse m6A modification patterns in STEMI.

### Immune microenvironment and biological pathways in the distinct m6A methylation subtypes

**Figure 6:**
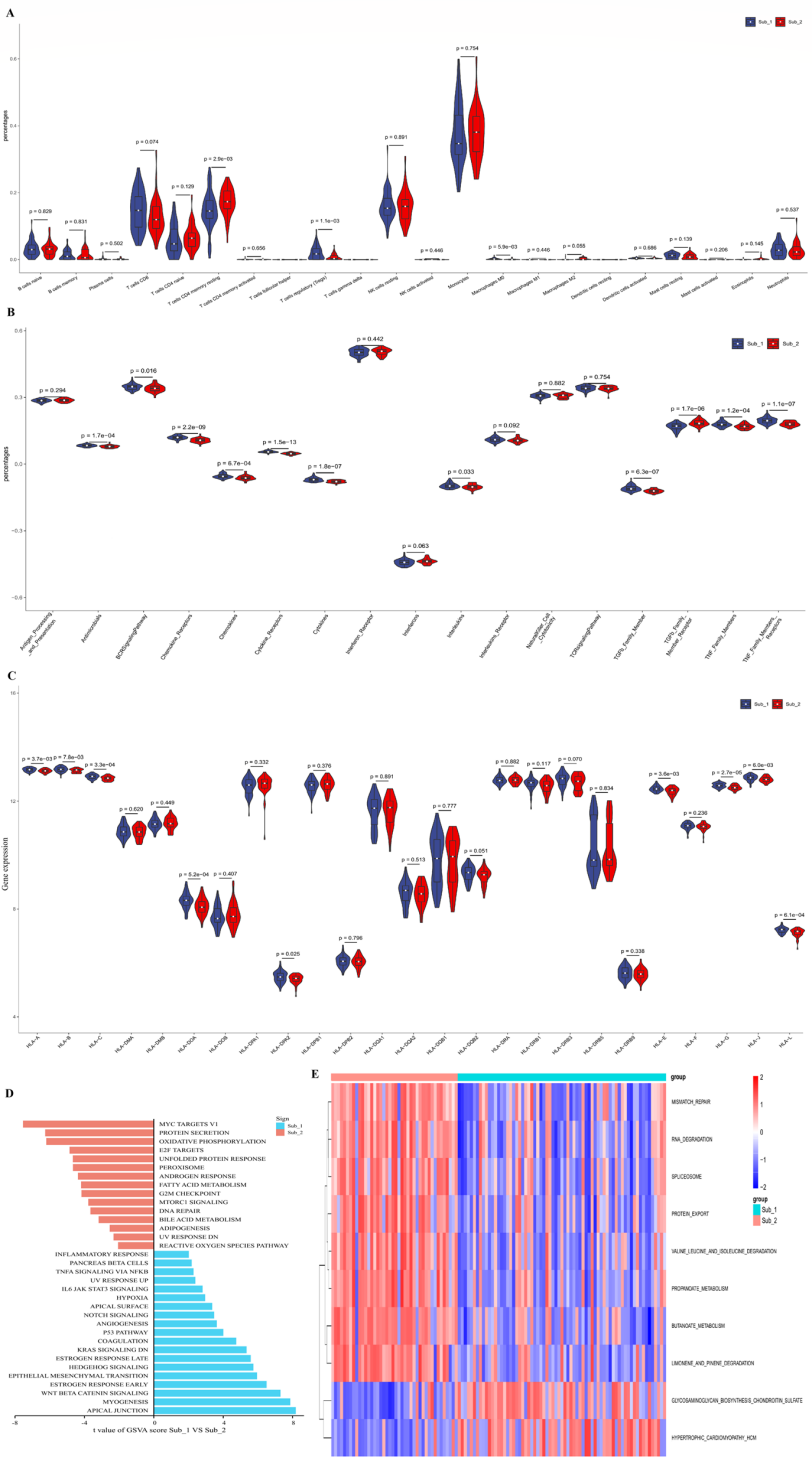
Diversity of immune characteristics in m6A subtypes and biological function. (**A**) Violin plot of all immune cell differentially infiltrated fractions. (**B**) Violin plot of the activity differences of immune reaction. (**C**) Violin plot of differential expression of 24 human leukocyte antigen genes. (**D**,**E**) Differences in the HALLMARKS pathway and KEGG pathway between the two distinct subtypes of STEMI.

To identify the differences in immune microenvironment characteristics between different m6A subtypes of STEMI, infiltrating immune cells, immune reaction activity and HLA genes were compared. We found that only three immune cells, cells, resting memory CD4 T cells, regulatory T cells, and M0 macrophages,, were different between the two m6A subtypes (Fig. 6A). For immune reaction activity, m6A methylation subtype 1 had a stronger immune response than type 2. There were 10 immune responses more active in subtype 1, while only the TGFb family member receptor pathway was very active in subtype 2 (Fig. 6B). Similarly, all differentially expressed HLA genes were highly expressed in subtype 1 (Fig. 6C). The immune differences between the two distinct m6A subtypes also suggested that m6A RNA methylation regulators had a crucial regulatory role in immune microenvironment of STEMI.

Then, GSVA was used to assess the biological functional pathways in two distinct m6A subtypes. Subtype 1 had more enriched pathways in immune related pathways, angiogenesis, coagulation and so on, whereas subtype 2 was more enriched in metabolism related pathways, such as protein secretion, oxidative phosphorylation, fatty acid metabolism, and adipogenesis (Fig. 6D,E).

### Identification of m6A mediated genes

**Figure 7:**
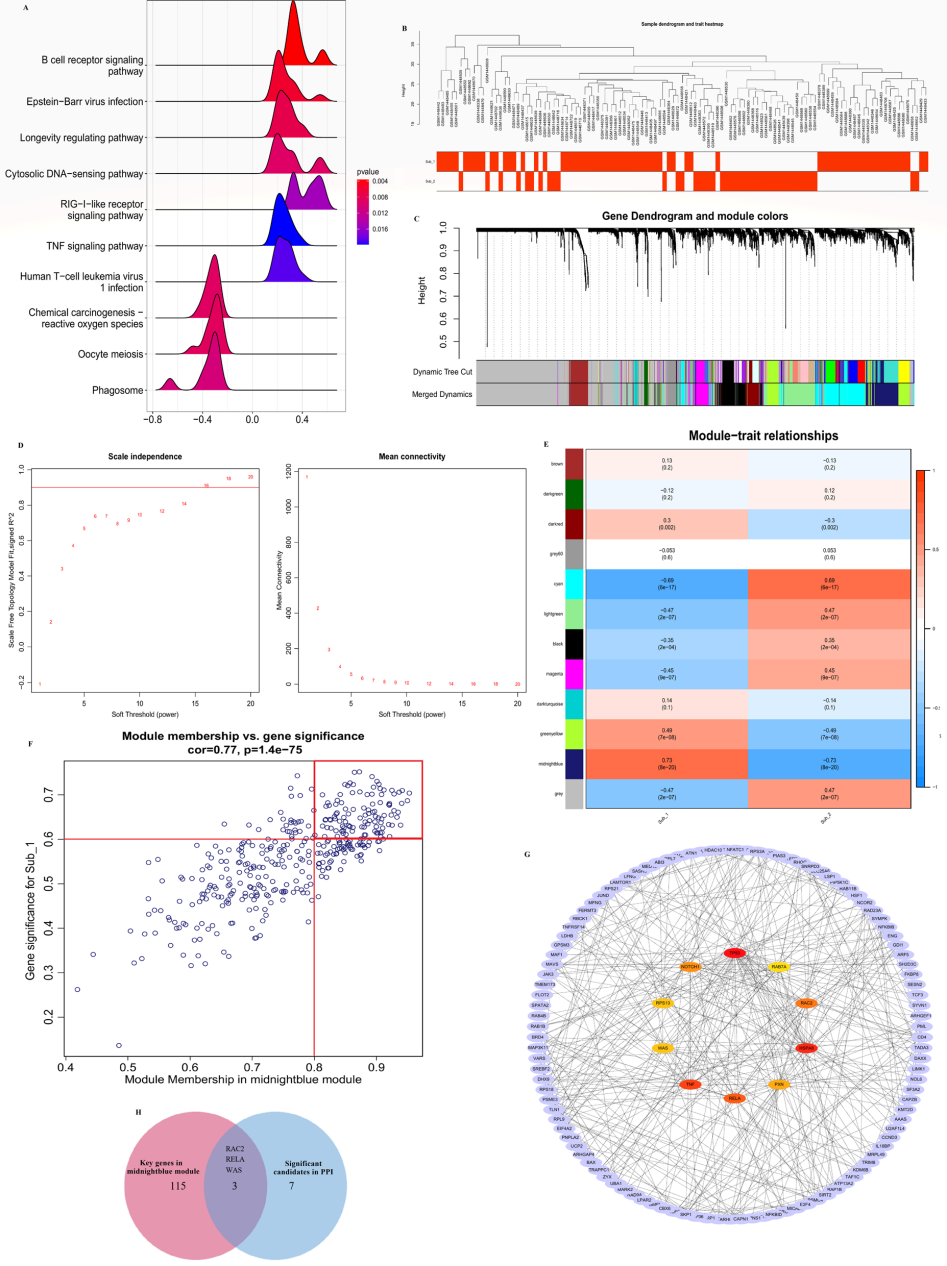
Identification of m6A methylation mediated genes by weighted gene coexpression network analysis (WGCNA). (**A**) Gene-set enrichment analysis of m6A phenotype-related genes. (**B**) Sample clustering diagram of two distinct m6A subtypes of STEMI. (**C**) Clustering dendrogram of m6A phenotype-related genes with dissimilarity based on topological overlap. (**D**) Scale independence and mean connectivity analysis. When the soft‐threshold β = 16, the correlation coefficient was greater than 0.9. (**E**) Module–trait relationships between the different m6A subtypes and module eigengenes. (**F**) Scatterplot of gene significance for m6A subtype 1 vs. module membership in the midnightblue module. The dots in the red box are key genes with module membership > 0.8 and gene significance > 0.6. (**G**) The protein–protein interaction network of the genes in the midnight blue module. (**H**) Venn diagram of overlapping genes from the significant candidates in the PPI network and the key genes in the midnightblue module.

To further investigate which genes were involved in the molecular mechanism of m6A methylation regulation, we identified m6A related genes and performed GSEA enrichment analysis (Supplementary 2). The results showed that m6A phenotype-related genes were mainly enriched in immune-related pathways, such as the B-cell receptor signalling pathway, RIG-I-like receptor signalling pathway and TNF signalling pathway (Fig. 7A). This finding also showed that m6A methylation can regulate the immune microenvironment in STEMI. WGCNA was used to identify key modules among different subtypes in STEMI. After performing hierarchical clustering analysis on the samples, correlation analysis was performed using the scale-free network module and different m6A subtypes (β = 16, Fig. 7B–D). The results showed that the midnightblue module was closely related to m6A subtype 1 (R = 0.77, P < 0.01, Fig. 7E). The genes with module membership (MM) > 0.8 and gene significance (GS) > 0.6 in the midnight blue module were regarded as the key genes (Fig. 7F). In addition, we imported the genes in the midnight blue module into the STRING database, constructed a PPI network via Cytoscape software, and identified 10 genes as significant candidates by ‘cytoHubba’ (Fig. 7G). Finally, we overlapped the significant candidates in the PPI network and the key genes in the midnightblue module, and 3 hub m6A phenotype related genes, RAC2, RELA, and WAS, were identified (Fig. 7H).

## Discussion

STEMI is one of the most dangerous diseases in clinical practice, and early prevention may reduce morbidity and mortality. Therefore, in recent years, researchers have been working to identify new biomarkers to improve the prediction and treatment of STEMI. RNA modifications play key roles in regulating molecular events and diseases^28^. Many studies have confirmed that immune cell infiltration is related to the occurrence and progression of STEMI. Many researchers are also actively studying the characteristics of the immune microenvironment in STEMI, which may have a significant beneficial impact on the long-term survival of patients with STEMI. M6A modification has the potential to broadly affect gene expression patterns and immune characteristics^29,30^. Studies have shown that the up- or downregulation of specific RNA m6A methylation regulators is associated with the occurrence of many diseases, while m6A modification mediates various molecular functions during RNA processing^31,32^. Moreover, m6A regulators are widely involved in the biological processes that promote tumour progression by regulating the immune microenvironment in multiple cancers and are associated with shortened survival^13,33,34^. Thus, it is unclear how m6A modification and its related modifier genes regulated in STEMI.

To better investigate these questions, we comprehensively analysed the role of m6A regulators in STEMI and revealed the relationship between m6A methylations and the immune microenvironment. First, we found that most m6A regulators were significantly differentially expressed between the STEMI and control groups and that there were substantial interactions between each m6A writer, eraser, and reader. Therefore, the biological function of m6A methylation regulators in STEMI may be the joint action of each effector. After LASSO and multivariate logistic analysis, we established a classifier and nomogram model based on 6 m6A regulators, which can easily distinguish the STEMI and control samples in the validation set and external independent validation set. The DCA curves suggested that the nomogram model may benefit STEMI patients. This finding also indicated that m6A methylation may play a crucial role in STEMI. Recent studies have found that ALKBH5 maintains angiogenesis in vascular endothelial cells after acute ischaemia, possibly due to its ability to reduce m6A methylation and downstream eNOS-AKT signalling^35^. FMR1 can protect cardiomyocytes from lipopolysaccharide-induced myocardial injury by regulating oxidative stress and apoptosis-associated factors^36^. Zhang et al. also found that the expression of KIAA1429 and YTHDF2 was upregulated in heart failure patients compared with that of healthy controls^37^. In general, few studies have explored the association of the six candidate m6A regulators with acute myocardial infarction, and further study of the mechanism is necessary in the future.

Then, we investigated the association of m6A methylation regulators with the immune microenvironment in STEMI, including immune cell infiltration, immune response activity, and the gene expression of HLAs. In immune cell infiltration, a positive correlation was found between YTHDC1 and resting memory CD4 T cells, while FTO was most negatively correlated with monocytes. Many studies have confirmed that the infiltration of resting memory CD4 T cells is closely related tokey genes in AMI^38–40^, but further research on m6A methylation regulation is needed in the future. Ke et al. found that in a mouse model of ischaemia‒reperfusion injury, FTO overexpression attenuated hypoxia/reoxygenation-induced cardiomyocyte apoptosis and inflammation, and FTO induced myocardial injury in a m6A-dependent manner^41^. Furthermore, altered expression of FTO was associated with m6A levels resulting in cardiac dysfunction after acute inflammation in rats^42^. The content of monocytes in the STEMI group was significantly higher than that in the control group. This finding was also consistent with the results of the immune infiltration analysis by Wu et al.^40^. Monocytes are recruited to the heart and trigger an intense immune and inflammatory response. Monocytes are pleiotropic cells of the innate immune system, and have a crucial effect on the initial immune response and myocardial repair after MI injury^43^. These findings enhance our insights into the m6A immune regulation mechanism in STEMI.

In addition, we obtained two distinct m6A RNA methylations based on the m6A expression profiles using the consensus clustering method. The active immune response activity and HLA gene expression were higher in m6A subtype 1 of STEMI, whereas, subtype 2 only had a higher abundance of infiltrating CD4 memory resting T cells and activation of the TGFb family member receptor pathway. GSVA enrichment analysis of the two subtypes found that subtype 1 was mainly enriched in immune-related pathways, including inflammatory response, TNF-α signalling via NF-KB, and IL6-JAK-STAT3 signalling, while subtype 2 was mainly enriched in metabolism-related pathways. The immune microenvironment of different subtypes also suggested the reliability of classification by different m6A regulators. It also helps us understand the underlying immune mechanisms of different STEMI subtypes, which can help to develop new targeted immunotherapies.

Finally, WGCNA was performed in the two distinct subtypes of STEMI samples and we found that the midnight blue module was associated with different m6A modification patterns, and further identified RAC2, RELA, and WAS, the three m6A methylation markers. RAC2 is a member of the small GTPases of the Rho family and can regulate the NADPH oxidase^44^. Several studies have confirmed that RAC2 affects the development of arteriosclerosis by regulating the activity of macrophages^45–47^. RELA, also known as NF-κB3 and p65, is a member of the NF-κB family. The NF-κB transcription factor family has important effects on innate and acquired immune responses and is a central mediator linking immunity and inflammation. The NF-κB signalling pathway has been confirmed to be associated with the occurrence and prognosis of acute myocardial infarction^48,49^. WAS is mainly involved in the formation of actin nucleation in vivo and plays a role in immune defence and homeostasis^50^. The mechanism by which WAS regulates acute myocardial infarction has not been studied, and it may be related to the regulation of T cells and B cells to play an immune role^51^. Based on the data available from previous studies, the three candidate m6A methylation markers are related to immunity, which again confirms the difference in immune characteristics between the two distinct subtypes.

Currently, epigenetic research in the field of acute myocardial infarction is limited and varied. The study of the m6A modification mechanism and immune microenvironment theory to explore the pathogenesis and treatment direction of acute myocardial infarction can fill the gap in epigenetic modification and immunity of acute myocardial infarction. Our study also found that the immune characteristics of STEMI patients were different, and immunotherapy for STEMI patients with different immune characteristics can effectively reduce the occurrence of major cardiovascular adverse events. However, our study also has some limitations that we must mention. First, in the Methods section, we analysed the m6A regulator and identified the immune microenvironment through bioinformatics, but did not confirm the relationship between the expression of key m6A genes and immune characteristics through a variety of different experimental methods. However, surprisingly, we obtained meaningful results in the joint evaluation of the ROC curve, qPCR and nomogram model. We will conduct further experimental research on this topic it in the future.. In addition, we did not have access to additional clinical data, such as cardiovascular-related risk factors, interventional therapy, and prognosis, which prevented us from correlating m6A patterns and other clinical features.

In conclusion, this study uncovered a potential regulatory mechanism of m6A methylation in STEMI, and a prediction model was built based on 6 m6A regulators. We further identified two distinct m6A subtypes with different immune microenvironments in STEMI, and 3 m6A subtype markers, all of which were immune-related genes. Thus, for m6A-based gene therapy, the development of m6A modulators or small molecular m6A agonists and antagonists, based on bioengineering materials, may have important clinical value in the treatment of STEMI. This study will help us to understand the underlying mechanisms of immune regulatory networks in STEMI and develop more effective immune-related treatments.

### Supplementary Information

Supplementary Information 1.

Supplementary Information 2.

Supplementary Figure 1.

## Data Availability

Publicly available datasets were analyzed in this study. This data can be found here: https://www.ncbi.nlm.nih.gov/gds/?term=GSE59867 and https://www.ncbi.nlm.nih.gov/gds/?term=GSE48060.
