# Supplementary material for: M6A regulator methylation patterns and characteristics of immunity in acute ST-segment elevation myocardial infarction
Source: Sci Rep. 2023 Sep 21;13:15688. doi: 10.1038/s41598-023-42959-5 (PMC10514189; doi:10.1038/s41598-023-42959-5)
Supplement: Supplementary file 3 — Supplementary Figure 1. [file 41598_2023_42959_MOESM3_ESM.docx]

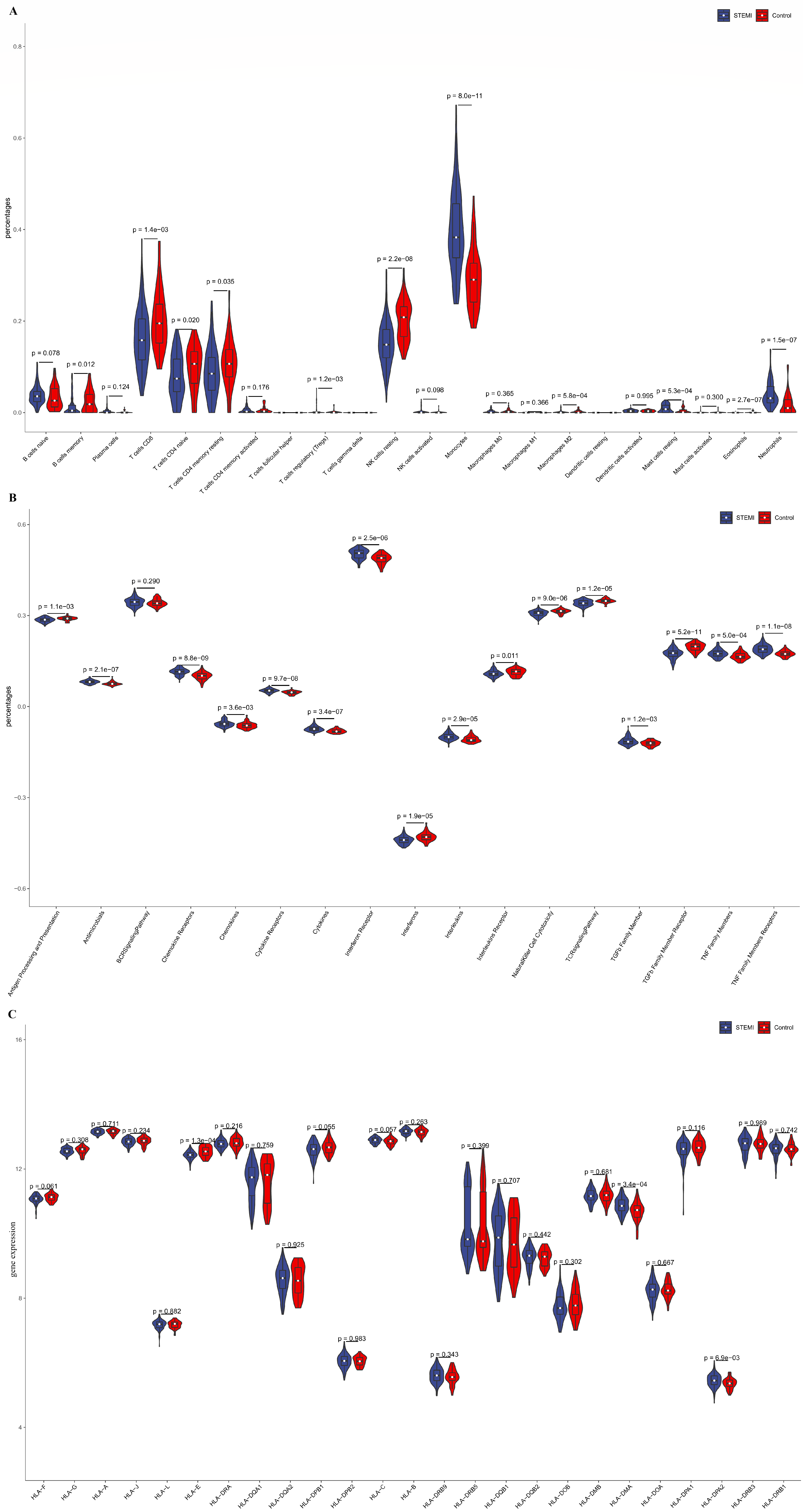


**Supplementary Fig. 1** Diversity of immune characteristics in the STEMI and control groups. (A) Violin plot of all immune cell differentially infiltrated fractions. (B) Violin plot of activity differences in immune reactions. (C) Violin plot of the differential expression of 24 human leukocyte antigen genes.
